# Engineering affibody-based biosensing platforms for cancer biomarker detection

**DOI:** 10.5599/admet.3363

**Published:** 2026-06-11

**Authors:** Zhafira Fauziah, Robeth Viktoria Manurung, Yuspian Nur, Dika Apriliana Wulandari, Salma Nur Zakiyyah, Irkham, Yeni Wahyuni Hartati

**Affiliations:** 1 Department of Chemistry, Faculty of Mathematics and Natural Science, Universitas Padjadjaran, Sumedang 45363, Indonesia; 2 Research Center for Smart Mechatronics, National Research and Innovation Agency (BRIN), KST Samaun Samadikun, Bandung 40135, Indonesia; 3 Study Center of Sensor and Green Chemistry, Faculty of Mathematics and Natural Science, Universitas Padjadjaran, Bandung 40132, Indonesia

**Keywords:** Electrochemical biosensor, optical biosensor, diagnostic

## Abstract

**Background and purpose:**

Cancer is one of the most life-threatening diseases and has the highest mortality rate worldwide. Delayed cancer diagnosis remains a major challenge in cancer treatment. Currently, conventional diagnostic methods still have several limitations, resulting in many cancer cases being detected only at advanced stages.

**Experimental approach:**

This review was conducted as a literature study of research on the development of affibody-based biosensors for cancer biomarker detection. The review discusses the characteristics of affibodies as bioreceptors, affibody synthesis methods, and their applications in electrochemical and optical biosensors.

**Key results:**

Various studies have shown that affibody-based biosensors exhibit high sensitivity and specificity for detecting cancer biomarkers such as human epidermal growth factor receptor 2, tumour necrosis factor-alpha, epidermal growth factor receptor, carcinoembryonic antigen and alpha-fetoprotein. The integration of affibodies into biosensor platforms enables low detection limits, wide linear ranges and good analytical performance in biological samples.

**Conclusion:**

Affibody-based biosensors show great potential as platforms for cancer diagnosis. The use of affibodies as bioreceptors offers several advantages over antibodies and aptamers, particularly ease of synthesis, high stability and strong binding affinity.

## Introduction

Cancer is a complex group of diseases characterized by the uncontrolled proliferation of abnormal cells that can invade surrounding tissues [1]. This uncontrolled growth results from accumulated genetic and epigenetic alterations that disrupt normal cellular mechanisms, including cell cycle regulation, apoptosis and DNA repair [2]. Globally, cancer remains one of the leading causes of morbidity and mortality. The dangers of cancer lie not only in the biological damage it causes, but also in the long-term quality of life of patients who experience a decline due to the side effects of therapy, such as extreme fatigue, chronic pain, immune system disorders and metabolic changes [3]. Furthermore, the high economic burden of cancer treatment adds to social stress [4].

Access to cancer diagnostic tools remains highly limited in developing countries. This limitation is one of the main reasons why 70 % of new cancer cases are diagnosed at an advanced stage. Consequently, opportunities for effective treatment are limited, leading to low patient survival rates. Detecting cancer at an early stage is one of the most critical factors associated with successful treatment outcomes [5]. Early cancer detection enables timely interventions and the possibility of less invasive treatments, resulting in more effective disease management and improved patient outcomes [6].

Cancer biomarkers serve as specific early indicators for early cancer detection and help guide therapy before the cancer progresses to an incurable stage. Furthermore, biomarkers are crucial for determining the likelihood of disease recurrence and for evaluating patient follow-up after chemotherapy, radiotherapy, or surgical treatment. Therefore, the development of more affordable and accurate diagnostic tools is essential, particularly in countries with limited healthcare resources [7]. Cancer biomarkers are generally detected using conventional techniques such as polymerase chain reaction (PCR) [8], western blotting [9], immunofluorescence [10] and liquid chromatography-mass spectrometry (LC-MS) [11]. However, these techniques have several major limitations, including high cost, relatively long analysis times, the need for specialized laboratory facilities and the requirement for trained personnel [12].

Up to the present time, many types of cancer remain difficult to detect at an early stage due to their non-specific symptoms and the limitations of conventional diagnostic methods [13]. This condition leads to most cancer cases being identified only at advanced stages, when the chances of successful therapy have significantly decreased [14]. Thus, the development of reliable, rapid and sensitive biomarker detection methods, especially for early-stage cancer, is urgently needed. In this regard, biosensors are promising candidates for the specific and simultaneous detection of multiple biomarkers, while also enabling analysis of associated biological interactions owing to their ease of modification [7].

Biosensors are integrated devices combining a bioreceptor and a transducer, capable of converting a biological reaction into a measurable signal that can subsequently be amplified and analyzed [14,15]. The biorecognition element plays the most critical role in a biosensor, as it determines the system’s selectivity and sensitivity through specific binding to the target analyte. Therefore, the selection of an appropriate bioreceptor is a crucial first step in biosensor design and development [14].

In biosensor systems, a major problem that often arises is non-specific binding. This can cause changes in the electrical signal, resulting in inaccurate measurement results. Therefore, the recognition element used must be highly specific to the analyte or target being detected. In this regard, amino acid-based proteins are well-suited for use as recognition elements. Among the various types of proteins, antibodies are the most widely used proteins for molecular recognition due to their highly specific ability to recognize targets [15]. However, in many cases, antibodies are not always available or cannot be produced. Even when available, antibodies may not have the appropriate specificity and affinity. Commercial antibodies also often exhibit poor characterization and variability between production batches [16]. On the other hand, the development of nanobodies (small antibody domains) for quantitative detection or simply for target capture is still relatively slow. Furthermore, both antibodies and nanobodies are produced through antigen immunization, which complicates their production and costs [17].

Due to various limitations in the use of antibodies, nucleic acid aptamers, typically 25 to 80 bases in length, were introduced as an alternative in vitro bioreceptor that can bind targets with high affinity and specificity [18]. This bioreceptor has several advantages, including long shelf life, resistance to heat and high temperatures, and can be modified without reducing binding affinity [19]. Therefore, aptamers are considered an alternative to monoclonal antibodies in many biotechnology applications, such as therapy and disease detection [20]. However, the development of aptamers through experimental methods such as systematic evolution of ligands by exponential enrichment (SELEX) requires significant time and energy and has limited reproducibility. In addition, aptamers have low resistance to nucleases, so they are not effective for clinical applications [18].

Recently, researchers have turned their attention to developing biologically neutral and physically stable protein scaffolds. One of the key advantages of this approach is that the scaffold protein design results in chemically uniform molecules that can be easily tailored to recognize a wide variety of analytes without requiring significant changes to the biosensor design or configuration. Furthermore, this approach enables a higher density of recognition elements on the biosensor surface, thereby improving sensor performance. In line with this approach, affibodies have emerged as a new class of engineered bioreceptors with superior target recognition characteristics [20].

Affibody molecules represent a new class of engineered affinity proteins with high affinity and specificity toward target proteins or peptides once isolated. These molecules are composed of α-helical structures and lack disulfide bonds, allowing their use in intracellular applications [21]. Unlike other bioreceptors, such as antibodies and aptamers, affibody molecules can be produced more rapidly *via* solid phase peptide synthesis (SPPS)due to their small size and rapid folding kinetics. Furthermore, these molecules can be produced recombinantly in bacteria such as *E. coli* [22]. Consequently, affibodies are considered a promising new alternative as affinity proteins, belonging to the class of scaffold proteins that mimic monoclonal antibodies but with enhanced properties [23].

Several previous review articles have discussed the use of affibody molecules in various application domains. Justino *et al.* [23] focused on the use of affibody in cancer cell targeting, affibody purification and medical imaging, without emphasizing early cancer detection. Meanwhile, Ståhl *et al.* [22] focused on the use of affibody for broader medical applications, including imaging and therapy for neurodegenerative diseases and inflammatory disorders. Then, Löfblom *et al.* [21] reviewed various affibody molecules that specifically target human epidermal growth factor receptor 2 (HER2) with their main focus on therapeutic applications, *in vivo* imaging and biotechnology. In contrast to the reviews above, this review offers a broader perspective, covering the detection of various types of cancer with affibody-integrated biosensor devices. This review explores the development of affibody-based biosensors for detecting various cancer biomarkers, emphasizing their applications in early diagnostics. Furthermore, this review examines affibody synthesis methods, such as solid-phase peptide synthesis (SPPS) and recombinant synthesis in *E. coli.* In addition, various signal amplification strategies, such as those used to improve detection performance, are discussed. Finally, this review provides a comprehensive overview of current applications and prospects in cancer biomarker diagnostics.

## Biomarker for cancer diagnosis

Cancer biomarkers, typically proteins, nucleic acids, or other molecular entities, are indicators of the presence, risk, or progression of cancer in an individual. Their detection and quantification are crucial for early diagnosis and treatment, as they provide specific information about a person's biological condition [24]. One type is the diagnostic biomarker, which is a biological marker used to detect or confirm the presence of a disease or specific condition in an individual, thereby facilitating earlier and more accurate diagnosis. There are also predictive biomarkers that can forecast a person’s response to medical therapy or to a particular environmental exposure. In addition, these biomarkers can help determine which therapy is most effective for a specific patient. Furthermore, therapeutic biomarkers help assess the effectiveness of pharmacological interventions and evaluate treatment safety and outcomes. Through these biomarkers, researchers and clinicians can monitor whether a drug is achieving its intended effect or causing unwanted side effects. Finally, prognostic biomarkers are used to estimate the likelihood of clinical events, disease recurrence, or disease progression among patients with a diagnosis [25]. A variety of commonly used cancer biomarkers are listed in Table 1.

**Table 1.** Overview of common cancer biomarkers used in clinical practice

Biomarker	Cancer type	Source	Clinical use	Ref.
Alpha-fetoprotein (AFP)	Liver	Blood	Staging	[26]
Cancer antigen 125 (CA125)	Ovarian	Blood	Monitoring	[27]
Carbohydrate antigen 15-3 (CA15-3)	Breast	Blood	Monitoring	[28]
Cancer antigen 19-9 (CA19-9)	Pancreas	Blood	Monitoring	[29]
Carcinoembryonic antigen (CEA)	Colon	Blood	Monitoring	[30]
Epidermal growth factor receptor (EGFR)	Non-small cell lung cancer	Tumour	Treatment selection	[31]
HER2/neu	Breast, gastric, esophageal	Tumour	Monitoring, prognosis and treatment selection	[32]
Prostate-specific antigen (PSA)	Prostate	Blood	Screening and monitoring	[33]

## Cancer biomarker detection methods

Various methods have been reported for the detection of cancer biomarkers, including PCR, enzyme-linked immunosorbent assay (ELISA), immunofluorescence, chemiluminescent immunoassay (CLIA) and liquid chromatography–tandem mass spectrometry (LC-MS/MS). Zhou *et al.* [34] developed a sandwich-based ELISA to detect the cancer biomarker carcinoembryonic antigen (CEA) using antibodies immobilized on the plate surface. Several types of cancer that can be associated with elevated CEA levels include colorectal cancer, pancreatic cancer, lung cancer, breast cancer, ovarian cancer, gastric cancer, thyroid cancer and several other cancers. The ELISA method in this study successfully detected the CEA biomarker with good linearity in the range of 2 to 64 ng mL^-1^ and achieved a limit of detection (LOD) of 2 ng mL^-1^. However, despite its reliable quantification, the conventional ELISA technique has a LOD only slightly below the nanomolar range, which is insufficient to meet clinical thresholds for many protein biomarkers, particularly in the early stages of disease.

Meanwhile, Park *et al.* [35] analysed HER2 mRNA expression in Formalin-Fixed Paraffin-Embedded (FFPE) breast cancer tissue samples using the reverse transcription quantitative polymerase chain reaction (RT-qPCR) method. In this approach, HER2 mRNA is first converted into complementary DNA (cDNA) *via* reverse transcription, followed by amplification with specific primers targeting the HER2 gene sequence. Detection is performed in real-time using fluorescent dyes such as SYBR Green or fluorophore-labelled probes (TaqMan probes), which generate signals proportional to the amount of amplified DNA. The results demonstrated that the RT-qPCR method achieved a sensitivity of approximately 93.0 % and a specificity of about 89.8 %.

Subsequently, Sekacheva *et al.* [36] reported the clinical validation of a CA-62 biomarker-based CLIA method for early detection of breast cancer. The CLIA method utilizes a light-emitting immunochemical reaction to measure biomarker levels with high sensitivity and specificity. This study demonstrated that the CLIA-CA-62 assay achieved a sensitivity of approximately 92 % and a specificity of 93 %.

Similarly, other advanced analytical methods, such as mass spectrometry-based approaches, also face practical challenges despite their excellent analytical performance. In a study by Chen *et al.* [37], a quantitative LC-MS/MS method was developed to measure alpha-fetoprotein (AFP) and core-fucosylated AFP in patients with hepatocellular carcinoma (HCC). This method demonstrated excellent performance with a LOD of 15.6 ng mL^-1^.

Conventional methods such as PCR, CLIA and LC-MS/MS indeed offer high sensitivity and accuracy. However, all three also have practical limitations, including the need for expensive instrumentation and equipment, trained operators, specialized reagents, complex sample-preparation procedures and controlled laboratory environments, which make them less suitable for point of care testing.

## Cancer biomarker-based biosensor

A sensor with a receptor specifically designed as a biological recognition element is called a biosensor. In 1962, the first generation of glucose oxidase (GOx) biosensors was introduced by Clark and Lyons, marking a turning point in biosensor development [38]. Biosensors are an excellent choice for detecting metabolic compounds and are widely used in clinical assays due to their high sensitivity, rapid response and low production cost [39]. This analytical device integrates biological recognition components with a physicochemical detector, consisting of a bioreceptor, transducer and detector with a digital output. Bioreceptors, including affibodies, antibodies, enzymes, aptamers and nucleic acids (DNA or RNA), selectively bind to their complementary targets, such as antigens, substrates and other DNA or RNA. Upon interacting with the target analyte, the bioreceptor generates a signal that the transducer processes for measurement. Transducers are classified into four types: electrochemical, optical, calorimetric and mass [40].

## Electrochemical-based biosensors

**Figure 1.**
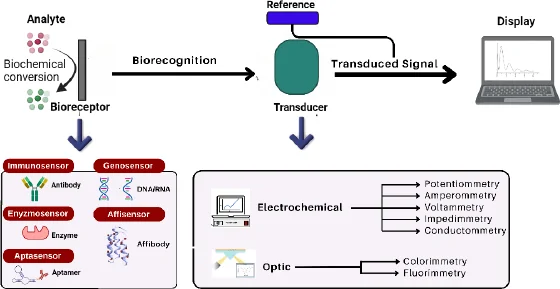
General scheme of an electrochemical and optical biosensor

Electrochemical biosensors offer high sensitivity and excellent selectivity for detecting targets using simple instruments and with fast reaction times (Figure 1). The analytical method using electrochemical biosensors is generally based on electron-transfer processes that occur at the electrode surface and on electroactive materials in the electrolyte [41]. Electrode surfaces coated with bioreceptors can specifically distinguish between biomolecules and targets, and the resulting interactions are converted into distinct electrical signals, enabling both qualitative and quantitative detection of targets [42]. The electrical signal generated from electrochemical measurements will be proportional to the analyte concentration [43]. Signal conversion can take the form of current, potential, impedance, or ion charge. Popular detection methods include cyclic voltammetry (CV), square-wave voltammetry (SWV), differential pulse voltammetry (DPV) and electrochemical impedance spectroscopy (EIS) [44,45]. There are three types of electrodes commonly used in electrochemical biosensors. The working electrode serves as the site of redox reactions and functions as a transducer, with its reduction potential depending on the analyte concentration. The reference electrode serves as a comparator to measure the potential at the working electrode, whose reduction potential is independent of the analyte concentration. The counter electrode conducts the entire current required to balance the current at the working electrode [46,47]. Numerous studies have explored the detection of cancer biomarkers using electrochemical-based biosensors, leading to significant advancements in their development. These innovations have enhanced the sensitivity, specificity and practicality of cancer biomarker detection, making electrochemical biosensors a promising tool for molecular diagnostics [40].

Cyclic voltammetry (CV) is one of the most influential analytical techniques that analyses the electrochemical properties of an analyte in solution [48]. CV is particularly useful for determining the standard oxidation and reduction potentials of biomarkers, which can indicate the presence of oral cancer markers such as cytokeratin fragment 19 (CYFRA 21-1) or IL-8 in saliva samples [49]. Several recent studies have demonstrated significant progress in the development of electrochemical biosensors for high-sensitivity, non-invasive detection of cancer biomarkers, particularly using CV techniques. For example, Shilpi Verma *et al.* [50] developed an electrochemical biosensor platform based on a ZnO-rGO nanocomposite for the non-invasive detection of IL-8 as an oral cancer biomarker. This detection system successfully identified IL-8 at low concentrations, ranging from 100 fg mL^-1^ to 5 ng mL^-1^, with a sensitivity of 12.46 ± 0.82 μA mL ng^-1^ and a LOD of 51.53 ± 0.43 pg mL^-1^. The synthesized nanocomposite exhibits high biocompatibility and excellent electron-transport properties, enabling its use in point-of-care (POC) applications for non-invasive onsite detection.

The label-free EIS approach offers a highly sensitive method for detecting a wide range of analytes, including cancer biomarkers, by monitoring changes in capacitance or charge-transfer resistance on a modified electrode surface [51]. In the study by Joshi *et al.* [52], the EIS technique was employed to detect two key cancer biomarkers: CEA and CYFRA 21-1. The detection mechanism is based on the interaction between oppositely charged antibodies and antigens, which reduces the overall charge of the complex. This change in charge density is subsequently transferred to the reduced rGO layer, altering the net charge in the p-type rGO layer and decreasing the electrical current passing through the rGO/MEL/antibody device. The sensor demonstrated the ability to quantify biomarker concentrations in saliva samples, with signal variation ranging from 7.14 to 59.1 % for CEA and 6.18 to 64.0 % for CYFRA 21-1, indicating a wide detection range and high sensitivity to changes in biomarker concentration.

In recent years, electrochemical biosensors have undergone extensive modification by integrating nanomaterials, enabling substantial improvements in signal amplification, LODs and assay specificity in cancer biomarker analysis. These enhancements are largely driven by engineered nanostructures that exhibit superior electron-transfer properties [52]. A high-sensitivity bead-based immunoassay with nanofluid preconcentration has been reported for biomarker detection as demonstrated by Fan *et al.* [53]. This approach employs a bead-based immunosensor integrated with a nanofluidic valve system, enabling real-time antigen quantification through microbiomolecule tracking velocimetry. With the ability to detect prostate-specific antigen (PSA) at an LOD of 50 pg mL^-1^ in just 20 minutes, this nanoparticle-enhanced platform demonstrates significant improvements in detection speed, sensitivity and the feasibility of multiplexed cancer biomarker analysis. The success of this system highlights how nanomaterials can substantially amplify biosensor performance.

Building upon this concept, Guo *et al.* [54] introduced a multiplex electrochemical immunoassay capable of simultaneously detecting AFP and CEA in human serum and saliva. Their design employs dual-wavelength quantum dots (QDs at 525 and 625 nm) supported by graphene as a conductive bridge, exploiting the strong signal amplification properties of QDs. The combination of streptavidin-coated CdSe/ZnS QDs and graphene facilitates robust electrochemiluminescent responses, enabling detection within an exceptionally wide dynamic range (0.001 to 0.1 pg mL^-1^) and achieving an ultralow LOD of 0.4 fg mL^-1^. This work further reinforces the crucial role of nanostructured materials in enhancing multiplexing capabilities and analytical sensitivity.

In a more advanced development, Kovarova *et al.* [55] reported a novel magneto-immunosensor that integrates electroactive nanocomposites to simultaneously quantify three ovarian cancer biomarkers: HE4, AFP and cancer antigen 125 (CA-125). This sensor combines multiple nanomaterial labels, including gold nanoparticles (AuNPs), CdTe QDs and PbS QDs, providing distinct electrochemical signatures for each analyte. Moreover, the incorporation of mesoporous silica nanoparticles (SiNPs) significantly boosts electrochemical output by increasing label-loading capacity. Compared to earlier designs, this system demonstrates how strategic nanomaterial engineering can expand multiplexing capability while further lowering LOD.

## Optical-based biosensors

Optical biosensors are classified into several categories, including colorimetric, fluorometric, luminometric, fibre-optic and surface plasmon resonance (SPR) biosensors. The performance of these optical biosensors continues to improve due to advanced structural designs and innovative biofunctional surfaces that resist nonspecific binding [56]. Among the various types of biosensors, fluorescence-based biosensors have emerged as a particularly prominent technology due to their distinct advantages. These characteristics result in robust biosensors capable of rapidly, accurately and specifically detecting targets in complex samples. The advantages of fluorescence biosensors include their non-invasive nature, ease of use and compatibility with various detection systems. The fundamental principle of fluorescence is that a molecule absorbs light at a specific wavelength (excitation) and then emits light at a different wavelength (emission) [57]. Biosensors utilize this property to generate a signal that correlates with the concentration of the target analyte. By measuring the fluorescence signal intensity, important information about the analyte can be obtained. This principle forms the basis of the specificity and sensitivity of fluorescence biosensors [58]. In recent years, fluorescence biosensors have developed rapidly. For instance, Zhao *et al.* [59] developed a fluorescence biosensing system based on two-dimensional molybdenum disulfide (MoS₂) for detecting the cancer biomarker CEA. The main sensing mechanism relies on MoS₂ nanosheets' ability to quench fluorescence signals via surface interactions and nonradiative energy transfer. Initially, the fluorescent probe interacts strongly with the MoS₂ surface, resulting in quenching of light emission *via* FRET or PET. When the target molecule is present, a specific complex forms that shifts the surface interaction equilibrium, causing the fluorescent probe to detach from MoS₂ and thereby restoring the fluorescence signal. The change in fluorescence intensity is proportional to the target concentration, enabling rapid and sensitive quantitative detection. The study successfully detected the CEA biomarker with a LOD value of 34 pg mL^-1^.

Colorimetric biosensors are practical detection devices that detect the presence and concentration of biomarkers *via* simple colour changes. The colorimetric method offers advantages for POCT and real-time monitoring due to its flexibility, simple operation, rapid results and versatility across many applications [58]. The basic principle of colorimetric assays is to detect the presence or absence of an analyte and its concentration through a change in colour or its formation. This colour change can be caused by dyes, enzymes, or nanoparticles (NPs) such as AuNPs. Colorimetric assays typically measure changes in light absorption (absorbance) or reflection (reflectance) resulting from chemical or biochemical reactions between the target analyte and a chromogenic probe. The resulting colour change generally arises from changes in optical properties, such as SPR, or from structural shifts. These colour variations can be observed qualitatively (with the naked eye by comparing colours) or quantitatively using reading devices such as scanners, cameras, smartphones, or spectrophotometers [60]. For example, Wang *et al.* [61] developed a wax-printing-based multilayer μPAD for the colorimetric detection of CEA. This method showed a wide linear range (0.5 to 70 ng mL^-1^) with a low LOD of 0.015 ng mL^-1^.

Advancements in optical biosensor modification have created significant opportunities to enhance both sensitivity and multiplexing capabilities for cancer biomarker detection. Various nanomaterial-based modification strategies, such as the incorporation of functionalized layers, the use of labelled nanoparticles, and the integration of microbeads as immobilization platforms, have been shown to markedly improve target-capture efficiency while amplifying the resulting optical signals [61].

For example, Liu *et al.* [62] developed a multiplex magnetic bead-quantum dot (QD) assay in a microarray format to detect lung cancer biomarkers, including CYFRA 21-1, neuron-specific enolase (NSE) and CEA. By employing magnetic beads and QDs conjugated with specific antibodies, their system successfully detected these biomarkers in serum samples at low concentrations, achieving LOD of 364 pg mL^-1^ for CYFRA 21-1, 38 pg mL^-1^ for CEA and 370 pg mL^-1^ for NSE.

Similarly, Di *et al.* [63] demonstrated multiple colorimetric strategies for cancer detection. They used modified AuNPs and antibody-conjugated exosomes in a nanozyme-assisted immunosorbent assay (NAISA) to detect exosomal proteins, including CD63, CEA, GPC-3, PD-L1 and HER2, from cell cultures and clinical serum samples. This approach allows differentiation of protein levels without additional labelling steps, resulting in a faster and simpler analytical workflow. Their findings confirm that the NAISA platform enables quantitative, highly sensitive detection of exosomal proteins, with a strong linear response over the concentration range of 13.75-220 μg mL^-1^.

## Affibody as an alternative bioreceptor for biosensors

Antibodies and aptamers are the two most commonly used bioreceptor types in biosensor development. Antibodies are large, Y-shaped proteins (~150 kDa) composed of heavy and light chains linked by disulfide bonds and generally contain glycosylated groups. Antibodies are produced by the immune system's response to specific antigens and are highly specific. However, antibodies have several limitations, such as poor stability to heat and temperature fluctuations, a relatively short shelf life and limited modification options that can reduce binding affinity [64]. Alternatively, aptamers are developed as synthetic bioreceptors consisting of small, single-stranded DNA or RNA molecules (6 to 30 kDa) obtained through an in vitro selection process using the SELEX method. Aptamers offer advantages such as good thermal stability, resistance to temperature variations, a long shelf life and ease of chemical modification without reducing binding affinity. However, aptamers also have disadvantages, particularly their susceptibility to degradation by nuclease enzymes in the blood [52,64,65].

**Figure 2.**
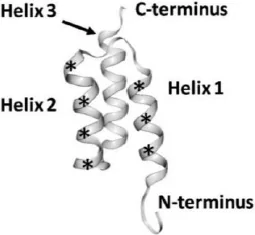
Structure of an affibody scaffold. Asterisks mark the segments where randomized amino acids are located. Reproduced from [66] with copyright permission

Due to the limited availability of antibodies and aptamers, affibody has emerged as a superior alternative bioreceptor for biosensor applications. Affibody is an alternative binding protein derived from the Z domain of Staphylococcal protein A [64]. It is a small protein (~7 kDa) composed of 58 amino acids arranged in an alpha-helical structure, as shown in Figure 2 [66].

Affibody acts as an immune-independent affinity molecule capable of targeting a wide variety of proteins [22]. Due to its alpha-helical composition, affibody lacks disulfide bonds, allowing its use in intracellular applications and enabling production in simple organisms such as prokaryotes rather than animal-based systems required for antibody synthesis [67]. A comparison of affibody, antibody and aptamer bioreceptors for biosensing applications is shown in Table 2.

**Table 2.** Comparison of affibody, antibody and aptamer bioreceptors for biosensor development.

Characteristics	Structure	Synthesis process	Other properties	Reference
Affibody	Small protein molecule (~7 kDa) consisting of 58 amino acids in alpha-helical form without disulfide bonds	Recombinant expression or SPPS	High solubility and stability under various conditions, strong binding affinity	[21,22]
Antibody	Y-shaped protein molecule (150 kDa) consisting of a chain of amino acids having heavy and light chains, disulfide bonds and glycosylation groups	Produced by the immune system in response to foreign substances	High specificity, short shelf life, not heat stable, temperature-resistant, limited modifications before losing binding affinity	[64]
Aptamer	short single-stranded DNA or RNA molecules (6 to 30 kDa) in the form of Secondary structures (loops, hairpins, stems) [22-24].	SELEX	Long shelf life, heat-stable, temperature-resistant, modifiable without decreasing binding affinity, easily degraded by enzymes in the blood	[19,68,69]

## Affibody synthesis methods

The ability of Affibodies to specifically recognize their targets is achieved through combinatorial protein engineering. Approximately 13 amino acids on the surfaces of helices 1 and 2 are randomized, and the best-performing variants are subsequently selected using high-throughput methods such as phage, bacterial, or yeast display. After selection using these various methods, affibody can be produced by chemical synthesis or by expression in *E. Coli* [70]. Meanwhile, affibody molecules can be produced recombinantly in bacteria such as *E. coli*, either as single domains or as more complex fusion constructs. This approach makes their production more cost-effective and simpler compared to antibodies [22]. In addition to recombinant production, affibodies can also be chemically synthesized using SPPS in a more controlled manner [71].

### Synthesis of affibody using E. Coli

*E. coli* is a bacterium frequently used to deliver recombinant proteins due to its rapid growth and the most studied biological characteristics [72]. Therefore, *E. coli* is a preferred host for affinity protein selection. In Gram-negative bacteria such as *E. coli*, a natural mechanism, the autotransporter system, provides an effective solution for displaying recombinant proteins on the cell surface [73]. This system has the primary advantage of efficiently transporting proteins across the inner and outer membranes sequentially, while simultaneously producing high levels of surface protein expression [74]. This high surface expression yields a strong signal when analysed by flow cytometry [75].

Recombinant affibody production in *E. coli* begins with inserting the gene encoding affibody into a suitable expression vector. This vector is then transformed into *E. coli* cells, converting the protein from a single gene. Once the plasmid containing the affibody gene is inside the cell, protein expression begins with transcription of DNA into mRNA, followed by translation of the mRNA into the affibody polypeptide chain by ribosomes in the cytoplasm. For surface display, an autotransporter system is used to carry affibodies from inside the cell out through the outer membrane and attach them to the cell surface [76].

### Synthesis of peptide-based affibody

Affibodies can also be produced through chemical peptide synthesis [77]. In SPPS, affibody synthesis is carried out by linking the first amino acid to a solid support (resin) *via* a stable covalent bond. The growing peptide chain remains bound to the solid phase and insoluble throughout the synthesis process, facilitating washing and filtration after each reaction step. This makes the synthesis of long peptides, such as affibody, more time-, labour- and material-efficient. Synthesis proceeds stepwise through cycles of deprotection of the α-amine group and subsequent attachment of the protected amino acid until the entire affibody sequence is assembled. Upon completion of synthesis, the peptide is released from the resin and purified in the solution phase [78].

Affibody molecules can also be specifically labelled at defined sites using peptide synthesis [66]. Their small size and rapid folding properties allow the incorporation of specific functional groups, such as reporter agents. Even with the addition of these functional moieties, affibodies remain much smaller than antibodies. In a recent study, Lindgren *et al.* [77] reported that SPPS of affibodies conjugated with fluorophores resulted in high yield and purity, enabling the rapid and straightforward production of multiple affibody variants.

Overall, both methods can produce affibodies with comparable structure and function. However, SPPS offers important advantages, including significantly lower endotoxin levels and higher product purity, making it more suitable for contamination-sensitive *in vivo* applications [79,80]. In addition, SPPS provides greater design flexibility, such as the ability to generate affibodies without a His-tag, thereby reducing molecular mass and enabling higher degrees of hydrogel modification [85]. Meanwhile, the recombinant method remains highly useful for early-stage screening or for producing larger proteins, since SPPS generally becomes less efficient as peptide length increases [81,82].

## Application of affibody-based biosensors in cancer biomarker detection

### Electrochemical biosensors

**Figure 3.**
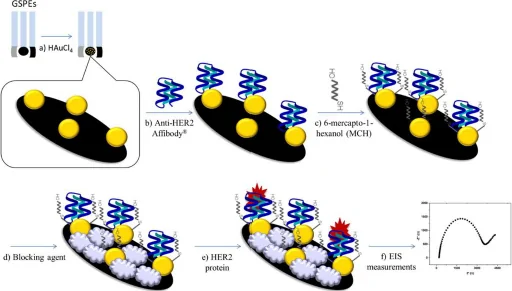
Schematic representation of the affisensor for HER2 detection (Reproduced from [84] with copyright permission)

In the study by Ravalli *et al.* [83], a label-free impedimetric affisensor for the determination of the cancer biomarker HER2 was developed, as illustrated in Figure 3 [83]. The procedure involved immobilizing anti-HER2 affibody molecules with terminal cysteine modifications on the surfaces of a screen-printed graphite electrode (SPGE) and a gold nanoparticle-modified SPGE (AuNPs-GSPE) *via* Au-SH bonding. After the formation of a self-assembled monolayer (SAM) using 6-mercapto-1-hexanol (MCH), non-specific binding sites were blocked with bovine serum albumin (BSA).

Although the use of BSA may form a thicker layer that can interfere with signal transmission, it effectively reduces non-specific protein adsorption by distancing inactive electrode areas from the sensitive region. Subsequently, affinity interaction with HER2 was evaluated using EIS. The developed biosensor exhibited a linear response range of 0 to 40 μg L^-1^ for HER2, with an LOD of 6 μg L^-1^. A good analytical response was also observed in serum samples spiked with HER2 protein. The kinetic and thermodynamic parameters of the affibody-HER2 affinity interaction were further analysed by SPR.

In comparison with the conventional HER2 detection methods typically based on antibodies, the impedimetric affisensor showed a fast, sensitive and specific detection of HER2 in real samples with a very low LOD (6.0 μg L^-1^), yielding promising results for the use of newly-engineered proteins in biosensor technology for clinical applications. Furthermore, the instrumentation of this biosensor has been miniaturized to pocket-sized dimensions, making it ideal for use in POC devices. The developed affisensor is label-free and designed for single-use, simplifying the detection process and preventing cross-contamination. However, this study also has limitations, namely the lack of direct comparison with standard clinical methods (ELISA/CLIA) on real patient samples to assess clinical relevance. Despite the simplicity of the label-free system, its use can make the signal highly dependent on the surface conditions of the sensor and sample, compared to labelled methods, whose signals are “stronger”.

**Figure 4.**
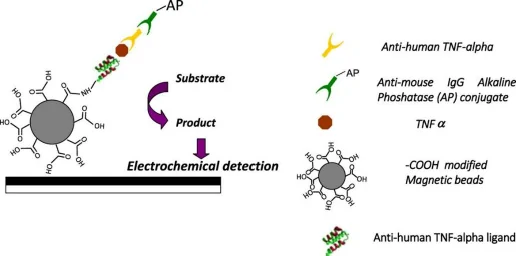
Electrochemical test scheme based on magnetic beads and disposable screen-printed sensors (Reproduced from [69] with copyright permission)

On the other hand, Baydemir *et al.* [69] developed a detection method for tumour necrosis factor-alpha (TNF-α) using affibody bioreceptors immobilized on magnetic beads (MBs), which served as capture agents in a sandwich assay configuration. Specific antibodies and secondary antibodies conjugated with alkaline phosphatase were employed, as illustrated in Figure 4.

The performance of this system was evaluated on standard solutions and human serum samples supplemented with TNF-α by DPV. A LOD of 0.038 ng mL^-1^ was obtained with a quantification range of 0.076 to 5.000 ng mL^-1^. Meanwhile, the measurement reproducibility, with a relative standard deviation of 7 %, indicated good performance. Unlike the study by Ravalli *et al.* [83], which employed a label-free system, this research utilized alkaline phosphatase (AP) to enhance the detection signal. MBs were also employed to facilitate both bioreceptor immobilization and immunocomplex separation. In addition, a comparison with a commercial ELISA method was performed to demonstrate the clinical relevance of the developed assay. The obtained Pearson correlation coefficient (*r* = 0.979) indicated that the method exhibited high accuracy and reliability in detecting TNF-α. The sandwich assay system used in this study also provided high sensitivity (LOD of 0.038 ng mL^-1^), making this study superior in sensitivity compared to other related studies.

On the other hand, although affibodies were used as bioreceptors, the antigen-capture step for TNF-α still relied on conventional antibodies, so the full advantages of using affibodies were not yet fully realized. Moreover, the enzyme label used in the system is chemically reactive, which may limit the assay's overall stability and practical applicability under real-world or field conditions.

**Figure 5.**
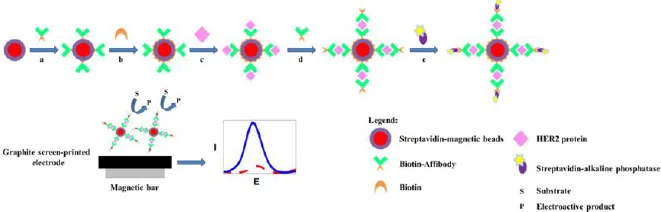
Schematic illustration of the streptavidin-modified MB-based affibody assay for HER2 detection (Reproduced from [85] with copyright permission)

Given the similarity of the test used, detection method and supporting elements, Ilkhani *et al.* [84] used the HER2 biomarker for breast cancer detection. They used a double-affibody (Af/Af) sandwich assay, in which the HER2 protein is captured between a primary bioreceptor immobilized on MBs (Biot-Af (Strept--MB/Af)) and a labelled secondary bioreceptor (Biot-Af). Detection was carried out by enzyme amplification by combining a streptavidin-alkaline phosphatase conjugate on Biot-Af, which converts the electro-inactive substrate into an electro-active product, as shown in Figure 5. The signal measured using DPV showed a LOD of 1.8 ng mL^-1^and a linear range of 0 to 20 ng mL^-1^. These results indicate that the Baydemir *et al.* [69] method is superior in sensitivity, whereas the approach of Ilkhani *et al.* [85] offers a broader range of target concentrations. Furthermore, the second configuration, the antibody/affibody sandwich assay (Ab/Af), utilizes an antibody (Ab1) immobilized on protein A-modified magnetic beads (ProtA-MBs). This method yields an LOD of 3.4 ng mL^-1^, an average RSD of 11 %, and maintains a linear response over the concentration range of 0 to 20 ng mL^-1^. The third configuration is the affibody/antibody sandwich assay (Af/Ab), which uses a biotinylated affibody as the capture receptor on Strept-MBs and a biotinylated antibody (Biot-Ab2) as the signal receptor. This assay has an LOD of 2.6 ng mL^-1^, an average RSD of 10 %, and a linear response in the range of 0 to 20 ng mL^-1^. Overall, the double-affibody assay (Af/Af) proved superior in sensitivity and reproducibility compared with the other configurations.

Among other studies, Ilkhani *et al.* [85] is the most relevant for HER2 (0-20 ng mL^-1^). This confirms the high potential of this method for real-world applications in the detection of HER2 cancer biomarkers. Furthermore, the affibody-only sandwich configuration offers higher selectivity and sensitivity than sandwiches incorporating antibodies. However, a limitation of this study is the lack of data on the long-term stability of the biosensor. This study also reported that affibody-modified magnetic beads could be stored at 4°C for approximately one week, without further testing for long-term bioreceptor durability.

### Optical biosensors

Pham *et al.* [86] explored the use of anti-HER2 affibody molecules conjugated with fluorescein isothiocyanate (FITC) for detecting the HER2 biomarker on HER2-positive extracellular vesicles (EVs) and compared their performance with conventional antibodies using the fluorescence polarization (FP) method.Anti-HER2 affibody molecules utilized in this work are head-to-tail affibody dimers linked through the peptide backbone for a higher binding affinity. In addition, the three anti-HER2 antibodies were derived from three different clones, namely 2G11 and 24D2, which were conjugated with FITC.

**Figure 6.**
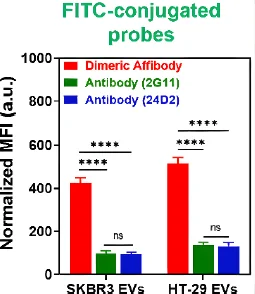
Superiority of FITC-conjugated dimeric anti-HER2 Affibody molecules over FITC-conjugated anti-HER2 antibodies in binding to SKBR3 EVs (Reproduced from [86] with copyright permission)

In this study, EVs were immobilized on MBs *via* antibody capture and subsequently detected with fluorescently labelled ligands. The results showed that FITC-bound dimeric anti-HER2 affibody could detect SKBR3 EVs and HT-29 EVs at low concentrations of 8.1×10⁶ and 7.0×10⁶ EV/mL, respectively. This LOD is much lower than the LOD of ELISA for EVs and many other published assays [87,88]. As shown in Figure 6, the two FITC-labelled anti-HER2 antibody clones did not display significant differences in binding to EVs from SKBR3 or HT-29 cells. In contrast, the dimeric anti-HER2 affibody demonstrated significantly superior HER2 receptor access, characterized by approximately 4.4-fold higher fluorescence signal on SKBR3 EVs and 3.8-fold higher on HT-29 EVs compared to conventional antibodies. Due to its small size (~6.5 kDa), the Affibody molecule may outperform monoclonal antibodies in accessing and detecting its targets on EVs.

On the other hand, the study by Sayyadi *et al.* [89] developed a simpler detection system that does not require two separate binding steps, unlike Pham *et al.* [86]. They directly used affibody-functionalized beads (affiBeads) as a novel strategy for high-sensitivity cancer exosome detection, using anti-EGFR affiBeads as bioreceptors. Detection was performed using a fluorescent detector with FITC labelling, which enhanced both the sensitivity and quantification of exosome detection.

**Figure 7.**
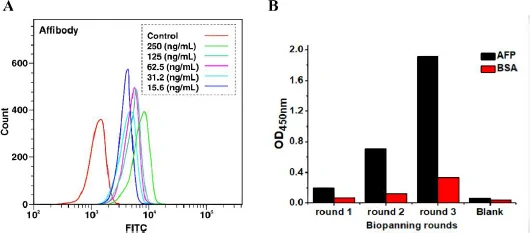
(A) cytometry histogram of affiBeads (Reproduced from [89] with copyright permission) (B) Biopanning rounds of phage ELISA (Reproduced from [90] with copyright permission)

During the fabrication stage, carboxyl-functionalized polystyrene microbeads were modified with anti-EGFR affibodies. The performance of the affiBeads in detecting exosomes *via* flow cytometry is shown in Figure 7a. A linear relationship was observed between the number of captured exosomes and the resulting fluorescence signal. Remarkably, even at very low concentrations (15.6 ng mL^-1^, approximately 12 exosomes per microbead), the affiBeads could still generate a significant signal.

Unlike commonly used conventional ELISA methods, Liu *et al.* [90] developed an affibody-based ELISA method. The affibodies used were synthesized using phage display technology. These affibody bioreceptors were used as capture reagents along with polyclonal antibodies to detect and quantify human serum proteins. This strategy was based on the affibody’s ability to bind to the solid phase at a higher molar density than antibodies due to its smaller molecular size.

After three rounds of biopanning (stepwise selection), the phage’s ability to bind AFP increased significantly, with the phage ELISA signal rising tenfold, as shown in Figure 7b. From the selected phage clones, ZAFP D_2_ was identified as an affibody with high specificity and strong affinity for AFP. To further enhance affinity, the researchers constructed a dimer form (ZAFP D₂)₂ by linking two ZAFP D₂ units using a G4SG4S linker.

**Figure 8.**
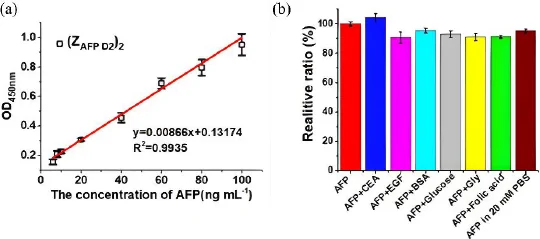
Results of the two-site ELISA method based on affibody-polyclonal antibody for AFP detection. (a) The linear range of the (Z_AFP D2_)_₂_ based two-site ELISA for the AFP concentration from 6 to 100 ng mL^−1^. (b) Selectivity of the (Z_AFP D2_) based two-site ELISA for AFP detection. (Reproduced from [91] with copyright permission)

The resulting dimer interacted with AFP twice as strongly as the monomer. The affibody covers specific regions of AFP, indicating shape complementarity that allows specific interactions. In this assay, ZAFP D_2_ or (ZAFP D₂)₂ served as the capture reagent binding to one side of AFP, while a polyclonal anti-AFP antibody acted as the detection reagent binding to the opposite side. An HRP-conjugated secondary antibody then produced the detection signal. This (ZAFP D₂)₂-based method demonstrated more sensitive results with a LOD of 2 ng mL^-1^ and a wide linear range of 6 to 100 ng mL^-1^, as shown in Figure 8a. Furthermore, selectivity tests, as shown in Figure 8b, demonstrated that even in the presence of various potential interfering agents (CEA, BSA, EGF, glucose, glycine, folic acid, PBS) at high concentrations, AFP detection remained stable, demonstrating the specificity of the test. Sample recovery using human serum ranged from 90 to 112 %, with good precision.

Compared to other studies, Liu *et al.* [90] highlighted the stability of the dimeric affibody (ZAFP D₂)₂, which remained stable even when heated to 80 °C. Moreover, the dimer exhibited twice the interaction strength with AFP compared to its monomeric form. The ELISA LOD based on (ZAFP D₂)₂ was four times lower than that of the monomeric ZAFP D2. In healthy adults, normal serum AFP levels are typically below 25 ng mL^-1^, whereas in patients with liver cancer, they can exceed 400 ng mL^-1^. Therefore, this LOD is well below the diagnostic threshold (10 ng mL^-1^), making this method suitable for detecting AFP in real serum samples.

Meanwhile, Zhang *et al.* [91] focused on detecting CEA, a biomarker for lung cancer. They covalently conjugated affibodies with PEG-functionalized gold nanoparticles to create a nanogold-affinity peptide probe as the biological recognition element. This conjugation provided more controlled orientation, better colloidal stability and more consistent performance compared to antibody-based systems.

**Figure 9.**
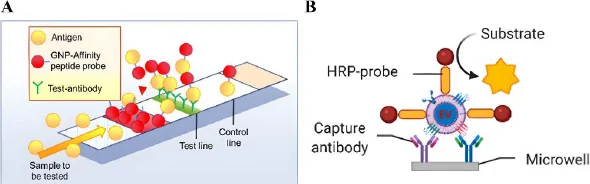
(A) Detection principle of the affibody nanogold probe test strip (Reproduced by [91]) (B) Illustration of the interaction between HRP-conjugated affibodies and immobilized EVs in microplate wells *via* CD9, CD63 and CD8 (Reproduced from [86] with copyright 2024 permission)

The detection mechanism of this device is shown in Figure 9a and follows a sandwich immunoassay format, in which the affibody and specific antibody work synergistically to capture the target antigen. The test strip membrane was coated with a CEA-specific antibody to capture the probe-antigen complex. The test results were determined by the colorimetric signal on the test line, allowing quick and easy interpretation. The system achieved a low LOD of 2.5 ng mL^-1^ and a diagnostic accuracy of 91.7 % in clinical serum samples.

In contrast, Pham *et al.* [86] used the enzymatic reaction between HRP and the TMB substrate to produce a colour change. HER2-positive EVs from SKBR3 cells were immobilized on streptavidin-coated wells using biotinylated capture antibodies (anti-CD9, CD63 and CD81), yielding an optical density (OD₄₅₀ nm) signal up to 10 times higher than antibody-based systems, as illustrated in Figure 9b.

This study also produced consistent results in human plasma samples, in which affibodies demonstrated high sensitivity with a LOD of 2.1×10^9^ EV mL-1, with EVs referring to extracellular vesicles. However, the HRP signal amplification system was less stable than inorganic nanoparticle labels, such as the colloidal gold system reported by Zhang *et al.* [91]. Various developments in cancer biomarker detection using affibodies are shown in Table 3.

**Table 3.** Affibody-based biosensor for cancer biomarker

Affibody	Bio-marker	Cancer type	Sensing layer	Signalling probe	Detection method	LOD, ng mL^-1^	Linear range, ng mL^-1^	Sample	Ref.
Anti TNF-α Affibody®	TNF-α	General	SPCEs functionalized HOOC-MBs	Alkaline phosphatase	DPV	0.038	0.076 to 5	Spiked serum	[69]
Biot-Af (Strept-MB/Af)+Biot-Af	HER2	Breast	Functionalized with strep-MBs	S-AP	DPV	1.8	0 to 20	Spiked serum	[85]
Ab1 (ProtA-MB/Ab)+Biot-Af						3.4			
Biot-Af+Biot-Ab2						2.6			
Anti-HER2 affibody	HER2	Breast	AuNP-GSPE	Label-free	EIS	6.0	0 to 40	Human serum	[84]
Anti-EGFR-affiBeads	EGFR	Lung	Functionalized with polystyrene carboxylate microbeads	FITC	Fluorescence (optic)	15.6	-	Diluted exosome	[89]
Nanogold-affibody	CEA	Lung	Immobilized CEA-specific antibody	AuNPs	Colorimetric	2.5	0 to 200	Human serum	[91]
(Z_AFP D2_)_2_	AFP	Liver	Two-site ELISA	HRP	Optic	2.0	6 to 100	Human serum	[90]
Affibody anti-HER2	HER2	Breast	Functionalized with biotinylated anti-CD9/CD63/CD81 antibodies	HRP	Colorimetric	2.1×10^9^*	10^8^ to 10^10^*	Human serum	[86]
			Functionalized with biotinylated anti-CD9/CD81 antibodies	FITC	Fluorescence polarization	8.1×10^6^*	10^8^ to 10*	Human serum	

*EV ml^-1^

## Challenge and future prospective

Although significant progress has been made in developing electrochemical biosensors based on affibody molecules, several fundamental challenges remain before this technology can be widely implemented in clinical settings. Affibody has lower production efficiency than other synthetic bioreceptors. Processes such as SPPS and expression engineering in conventional E. *coli* systems still require specialized chemicals, multistep processing and lengthy optimization times. Furthermore, phage display methods require large, complex phage libraries and the selection of highly hydrophobic affibodies can compromise stability and specificity. Another limitation is that affibodies displayed on the phage surface do not always retain the same properties as recombinant proteins, leading to differences in affinity and specificity in practical assays. Therefore, strategies are needed to make affibody synthesis more affordable, efficient and scalable.

One promising solution is the use of low-cost microbial expression systems such as *E. coli* strain BL21(DE3) or *Lactococcus lactis* combined with auto-induction medium techniques. This technique eliminates the need for manual induction control and reduces production costs by up to 40 %. Furthermore, the application of cell-free protein synthesis (CFPS) is a promising new trend because it enables rapid affibody production (within hours), without the need for cell culture. This technology is well-suited for on-demand or custom-made affibody production, for example, to detect rapidly emerging new targets such as new virus variants. Research by Lindgren *et al.* [77] showed that optimizing SPPS using microwave-assisted synthesis can reduce synthesis time by up to 60 % without compromising yield. Meanwhile, Wagner *et al.* [92] reported the efficient and economical production of affibody through an *E. coli* lysate-based cell-free synthesis system, yielding functional protein in less than 2 hours. However, the affibody synthesis process had some drawbacks. The phage display method required a large and complex phage library. In addition, selecting highly hydrophobic affibodies could affect stability and specificity. Another limitation was that the affibody displayed on the phage surface did not always retain the same properties as the recombinant protein, potentially leading to differences in affinity and specificity in practical assays.

From a molecular design perspective, miniaturizing binding domains is also an important strategy. By performing in silico design and molecular docking simulations, the binding time of affibodies can be optimized for shorter periods, thus reducing raw material costs and synthesis time.

Furthermore, integration with automated bioconjugation and synthetic microreactors enables parallel synthesis at a microscale (microfluidics-assisted peptide synthesis). This approach can accelerate the synthesis process, avoid wasting time and save expensive solvents and reagents, such as coupling agents (*e.g.* HATU or DIC).

Green chemistry approaches are also beginning to be implemented, for example, replacing toxic organic solvents (DMF, DCM) with environmentally friendly solvents such as deep eutectic solvents (DES) or water-ethanol, thus not only reducing waste costs but also increasing the downtime of the synthesis process.

The integration of affibody into microfluidic chips and portable biosensors is also an important direction for developing rapid, user-friendly POC systems. This combination also enables the integration of multiple functions in a single device, such as sample processing, separation, target recognition and signal reading, simultaneously, forming an integrated diagnostic system based on a lab-on-a-chip.

Meanwhile, in real-world applications, affibody often suffers from nonspecific binding to other proteins or matrices, which reduces the signal-to-noise ratio. Therefore, improving specificity is challenging. Computational approaches, including in silico protein design and structural simulations, can accelerate the selection of mutations that improve the constellation of interactions at the binding site without compromising molecular stability.

## Conclusion

Affibody-based biosensors have emerged as powerful devices for the sensitive and specific detection of cancer biomarkers, offering remarkable potential across a wide range of diagnostic applications. This technology exhibits exceptionally high sensitivity, with very low LODs, making it ideal for clinical diagnostic use. The integration of affibody molecules into both electrochemical and optical sensing platforms has significantly enhanced the overall performance of these biosensors.

This review highlights significant progress in the application of affibody-based biosensors for detecting various cancer biomarkers, including TNF-α, HER2, EGFR, CEA and AFP. Among the methods discussed, the electrochemical affibody biosensor developed by Baydemir *et al.* [69] stands out as one of the most effective, demonstrating high sensitivity and specificity for TNF-α detection. This method employs screen-printed carbon electrodes (SPCEs) and MBs as a support for affibody immobilization. The use of magnetic beads accelerates detection time and minimizes matrix effects, achieving a LOD as low as 0.038 ng mL^-1^. Furthermore, the optical affibody biosensor described by Zhang *et al.* [91] also offers excellent sensitivity and selectivity for detecting the CEA biomarker. This approach utilizes PEGylated gold nanoparticles (AuNPs) in a test strip format and achieves a LOD of 2.5 ng mL^-1^, surpassing the clinical threshold of 5 ng mL^-1^.

Despite these significant advancements, several challenges remain for the wide-scale implementation of affibody-based biosensors. Issues such as sensor stability, scalability, production efficiency and non-specific binding still require further optimization. Nevertheless, the progress achieved thus far underscores the tremendous potential of this technology to revolutionize molecular diagnostics and precision medicine. As research continues to advance, these biosensors are expected to become more accessible, more cost-effective and more reliable, ultimately serving as valuable tools for early disease detection, personalized therapy and global health monitoring.
